# The use of race and ethnicity in sickle cell disease research

**DOI:** 10.1186/s12874-025-02513-5

**Published:** 2025-03-07

**Authors:** Aida S. Kidane Gebremeskel, Minke A. Rab, Erik D. van Werkhoven, Teun B. Petersen, Marjon H. Cnossen, Amade M’charek, Karlijn A. C. Meeks, Anita W. Rijneveld

**Affiliations:** 1 Department of Pediatrics, Pediatric Hematology, Erasmus Medical Center, Dr. Molewaterplein 40, Rotterdam, 3015 GD The Netherlands; 2 Section on Social and Cognitive Developmental Neuroscience, Intramural Research Program, National Institutes of Mental Health, 9000 Rockville Pike, Maryland 20892 Bethesda, USA; 3 Department of Hematology, Erasmus Medical Center Cancer Institute, Dr. Molewaterplein 40, Rotterdam, 3015 GD The Netherlands; 4 Central Diagnostic Laboratory-Research, University Medical Center Utrecht, Heidelberglaan 100, Utrecht, 3584 CX The Netherlands; 5 Department of Biostatistics, Erasmus Medical Center, Dr. Molewaterplein 40, Rotterdam, 3015 GD The Netherlands; 6 Department of Epidemiology, Erasmus Medical Center, Dr. Molewaterplein 40, Rotterdam, 3015 GD The Netherlands; 7 Department of Cardiology, Thorax Center, Cardiovascular Institute, Erasmus Medical Center, Dr. Molewaterplein 40, Rotterdam, 3015 GD The Netherlands; 8 Anthropology, University of Amsterdam, Nieuwe Achtergracht 166, Amsterdam, North Holland 1018WV The Netherlands; 9 Center for Research on Genomics and Global Health, National Human Genome Research Institute, National Institutes of Health, 12 South Drive Bldg 12A ste 1025, Bethesda, MD 20892-5611 USA

**Keywords:** Ethnicity, Race, Sickle cell disease, Confounder adjustment, Ethno-racial categories

## Abstract

This study explores practices surrounding the operationalization of ethno-racial categories (ERCs) as confounders in biomedical research, with a focus on sickle cell disease (SCD) as a model. ERCs, often aggregate labels encompassing diverse individuals which raises questions about their relevance as confounders. Given SCD’s racialization as a “Black” disease, understanding ERC utilization is crucial. This study analyzed 1,105 SCD studies published globally. Data were collected on whether ERC adjustment was employed, regional variations in ERC-adjustment rates, labels used for ERCs, rationales provided for ERC matching, and methods used for ERC determination. 28% of the studies utilized ERC adjustment, with significant regional disparities (*p* < 0.001). Notably, Western studies showed higher rates of ERC adjustment compared to other regions. However, crucial details such as ERC labels and methodology were frequently missing. Commonly used labels included “African” or “Black.” Only 7% of studies provided explicit rationales for ERC matching, and 70% did not specify the method used for ERC determination. The findings underscore the need to adhere to guidelines on ERC operationalization in biomedicine. The lack of standardized practices raises concerns about potential biases and misinterpretations in research outcomes. Adhering to clear guidelines can mitigate the risk of perpetuating racial stereotypes and inequalities while ensuring research integrity.

**Supplementary Information:**

The online version contains supplementary material available at 10.1186/s12874-025-02513-5.

## Clinical trial number

Not applicable.

**Supplementary Information:**

The online version contains supplementary material available at 10.1186/s12874-025-02513-5.

**Key terms**:

**Table:** 

ERC	Ethno-racial category
SCD	Sickle Cell Disease
One-country studies	Studies conducted within a single country, with a sample of the population based in that country
Cross-national (comparative) studies	Studies using study populations from different countries
Operationalization of race and ethnicity as confounders	The way very contextualized concepts such as race and ethnicity are being put to use as confounders (i.e., controlled for in the analysis)

## Introduction

Race and ethnicity are demographic categories often used in biomedical research. Especially in areas that have been referred to as “the Western world,” they serve as increasingly important axes along which differences in health outcomes are stratified [1]. Biological, socioeconomical, and other environmental exposures play a role in creating and perpetuating (racial) health disparities. ERCs are contingent upon time, place and sociopolitical context. Therefore, unlike other demographic categories such as age and sex assigned at birth, ethno-racial categories (ERCs) have proved hard to harmonize internationally [2]. For example, the label “Black” in the UK versus South Africa likely includes individuals or groups with distinct genetic backgrounds, varying access to resources, and exposure to different environmental variables that influence their health. In highly admixed populations, such as in Brazil, operationalizing ERCs as biological proxies, poorly translates to genetic ancestry [3]. However, biomedical research studies appear to give the contextual nature of ERCs little consideration [4, 5]. 

Reporting on race- and ethnicity-based differences without context, can lead to unintended social and biological reification of these population descriptors. Since the rise of the Black Lives Matter (BLM) movement and health disparities accentuated by the COVID-19 pandemic, medical journals are increasingly discussing this topic [6, 7]. A recent systematic analysis of UptoDate^®^ articles demonstrated the biologicalization of race in 93·3% of all documents [8]. “Black race” was assumed to correlate with genetics or clinical phenotype, discarding race as a social determinant of health. Aside from being used as demographic descriptors, ERCs are also sometimes considered as confounders and therefore included in the analysis of a clinical study.

Whether an ERC is a confounder, depends on the research question [8, 9]. In particular, when considering an ERC as a confounder instead of a mere demographic descriptor, providing context to clarify its association with the study outcome, becomes crucial. Several systematic reviews on the use of ERCs in major epidemiological journals, described that 29% of 329 studies that used ERCs and published between 1995 and 2018, provided a rationale for their use. ERCs used as analytical variables also enter worldwide clinical practice as correction factors in clinical race-adjusted algorithms [5]. A well-known example is the “Black” race correction factor in estimated glomerular filtration rate (eGFR), which has been used in clinical practice worldwide [7]. However, the validity of this eGFR formula, which was originally derived from a study involving Black American participants, becomes more questionable when applied to individuals not identified as African or Black American. These resignations have recently led to the publication of recommendations that suggested removing Black race as a factor in the eGFR formula and the most recent eGFR formula is indeed “race-free.” [10–13].

Up until recently, the decision of whether and how to report on race and ethnicity in biomedical literature was at the discretion of the authors. However, publishers have put forward recommendations advising authors to exercise care and consideration in reporting race and ethnicity. Without offering a standard approach, these guidelines underline the importance of describing categorization methodology, and interpreting race and ethnicity-related study results [14–16]. Furthermore, The National Academies of Sciences, Engineering, and Medicine (NASEM) also published guidelines on the operationalization of ethnicity and race in biomedical research. Researchers should justify their use of ethnic and racial categories (ERCs), remain transparent, and critically evaluate their approach. Key recommendations include disaggregating data, identifying confounding factors often conflated with ERCs, and adjusting study designs accordingly [17]. 

In Western countries, a historically racialized disease is sickle cell disease (SCD). SCD is a hereditary hemoglobinopathy characterized by the formation of dysfunctional erythrocytes [18]. The two hallmarks of SCD are increased hemolysis that results in chronic anemia and vaso-occlusion, resulting in painful episodes and multisystem organ damage [18]. SCD patients experience an average life expectancy of 54 years in high-resource countries, as well as life-long disabilities [19]. Approximately 90% of the SCD patient population lives in three countries: Nigeria, India, and the Democratic Republic of Congo [20–22]. The prevalence of SCD is mostly limited to malaria-endemic regions or the diaspora of these areas, since carriership of the HbS gene confers a survival advantage when infected with *Plasmodium Falciparum* malaria. In the United States and Europe, it is estimated that SCD prevalence is three per 10,000 individuals and one per 10,000, respectively. Outside Europe and the USA, the significance and relevance of ERCs may differ, as the geographical survival advantage of the HbS gene tends to include various ethnic groups living malaria-endemic areas [23, 24]. In the Western context, SCD researchers are faced with a case group racialized as “non-white” [25]. These SCD patients often have a migration background from Sub-Saharan Africa or are descendants from victims of the transatlantic slave trade. Outside Europe and the USA, the significance and relevance of ERCs may differ, as the survival advantage of the HbS gene tends to include various ethnic groups living inside these malaria-endemic areas. This context could dilute the relevance of including specific ERCs in SCD research from these countries.

Historically, individuals with SCD have faced structural discrimination across multiple domains. In science and science policy, insufficient research funding has hindered progress, delaying the development of novel treatments [26]. In American clinical care, there is a considerable shortage of comprehensive care centers for SCD, which would have the capacity to drastically improve care outcomes by providing holistic care [27]. Furthermore, stigmatization and scrutiny from healthcare professionals are widespread problems, for example around opioid use during pain crises [28, 29]. This is compounded by the fact that SCD symptoms and associated struggles are often not visible to others [30]. Beyond science and healthcare, SCD patients experience interpersonal discrimination based on race and disability, leading to systemic inequities [31]. These include educational disadvantages, employment discrimination, and gaps in insurance coverage, which collectively restrict access to high-quality healthcare on an individual level [29, 32–35]. 

All in all, individuals with SCD face significant marginalization, and science must not exacerbate this. The process of essentialization, which reduces complex identities to fixed traits, underpins and reinforces discrimination and is a risk when using race and ethnicity uncritically. Using race-adjusted kidney function estimators, for instance, might overestimate kidney function in individuals racialized as black affected by SCD nephropathy. This might potentially delay referrals to specialist care or consideration for kidney transplantation [7, 36]. Researchers must approach the use of ERCs carefully to prevent reinforcing biases and inequities.

SCDs’ high global prevalence spans diverse ethnicities and races. Furthermore, as a multisystem disorder, its impact has been studied across various clinical specialties. These characteristics make SCD an exemplary case for investigating the practice of ERC operationalization as a confounder in biomedical research from a global perspective. It is unknown whether this diversity of ERCs, contained by the SCD patient population is accounted for in biomedical research.

In this study, we analyze patterns about the use of ERCs as confounders (i.e., that ERCs were controlled for in the analysis) in SCD research. Furthermore, possible influences surrounding confounder adjustment of ERCs are explored. We set out to determine the prevalence of ERC-confounder adjustment and its correlation with the adjustment for other covariates. Furthermore, we explore if and how ERCs are contextualized for use as covariates in SCD research publications. As we stand at an important juncture in reporting racial and ethnic categories in the field of biomedicine, this retrospective analysis serves as a critical baseline measurement for evaluating race and ethnicity as categorical constructs in this field.

## Methods

### Search strategy and screening process

**Fig. 1:**
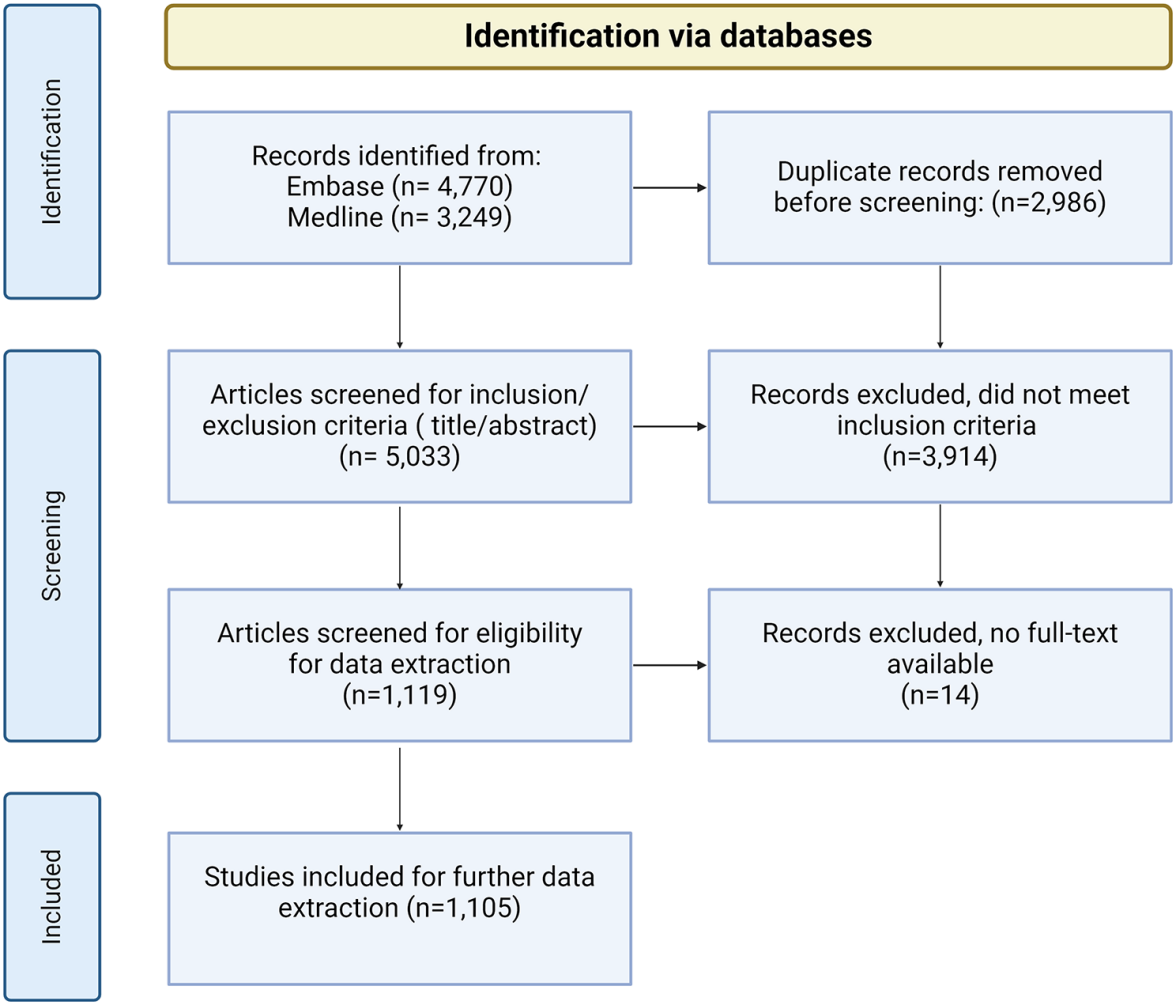
PRISMA ™ flow diagram of the selection process for the quantitative and qualitative literature analysis of SCD research. Exclusion criteria were articles published before January 1, 2011 of after November 8, 2022, non-English manuscripts, designs other than case-control, and letters or brief reports. Figure created with BioRender

We systematically searched for original, peer-reviewed publications in Embase (via Ovid) and MEDLINE (via PubMed) published between January 1, 2011 and November 8, 2022. The search strategy was created in collaboration with information specialists. The following keywords were used for this search: “sickle cell,” together with descriptions of (specific parts of) study designs, such as “cohort analysis” and “control group” (Supplementary Table 1). This search yielded 5,033 results after the removal of duplicates. Inclusion criteria were original research in the English language and a comparison of cases with controls, where cases are individuals diagnosed with SCD and the controls were not. We focused on case-control studies to isolate instances where authors had the opportunity to make deliberate choices regarding ERC adjustment. Records were excluded if they were letters, abstracts, or brief reports. Exclusions were independently screened by two researchers. Any articles where there was uncertainty about inclusion or exclusion were reviewed and discussed by the research team. This resulted in 1,105 articles which were used for data extraction (Fig. 1). The majority of the selected studies consisted of one-country studies (*n* = 1,085), i.e., studies conducted within one single country, with the study population originating from that country. For this analysis, complicated contexts such as: cross-national studies (*n* = 20), studies including Sub-Saharan African populations and publications that mention multiracial or multi-ethnic individuals, were described separately.

### Data extraction

The following outcome data were extracted: year of publication, country, whether confounder adjustment took place based on age, gender or sex, socioeconomic status, ERCs or other categories, whether an explanation was provided for ERC-confounder adjustment, labels used for ERCs, methods used to determine the ERCs of study participants and whether participants with a mixed racial or ethnic background were annotated. The citation rate and CiteScore percentile per article were extracted from Scopus on the 2nd of March in 2023.

#### Global regions

The one-country studies were categorized into global regions. We separated high-resource world regions, such as Europe and North America, from others because of the distinct challenges faced by racial and ethnic communities in these settings these “Western contexts”. The Caribbean were considered a separate region because of the self-identification of SCD patients as Caribbean. The South Asian Region only represented SCD studies with an Indian study population. There were no eligible studies from other world regions such as East Asia and Oceania. For an overview of the grouping of countries of origin of the various studies under specific geographical regions, see Supplementary Table 2. Supplementary Table 3 provides an overview of the number of studies per country of origin.

### Data analysis

Prior to analysis, data preprocessing and cleaning steps were performed. Associations between the manuscript being published in a Q1 journal (yes/no), (non-) demographic covariates and the country of origin of the manuscript were analyzed using chi-square tests, or the Cochran-Armitage Trend Test for ordinal variables. Binomial Generalized linear mixed models were used with a logit link function (with R package lme4 version 1.1.32) to examine factors associated with ERC adjustment (yes/no). Fixed effects were presence of adjustment for socioeconomic status (SES), adjustment for other potential confounders, and the geographical region from which the study participants were recruited. The journal was entered as random effect. Analyses were performed in R (version 4.2.3) and the package ggplot2 was used for visualization purposes [37]. 

## Results

### Characteristics of reviewed literature

Among the 1,105 included articles, 1,085 were single-country studies, and 20 were cross-national studies. The countries the study population was sourced from, are presented in the Supplementary material. The dataset and the analyses can be accessed on GitHub via the following link: https://github.com/AidaasinAyda/SCD_conf_correction.

### Prevalence of ERC-confounder adjustment

**Fig. 2:**
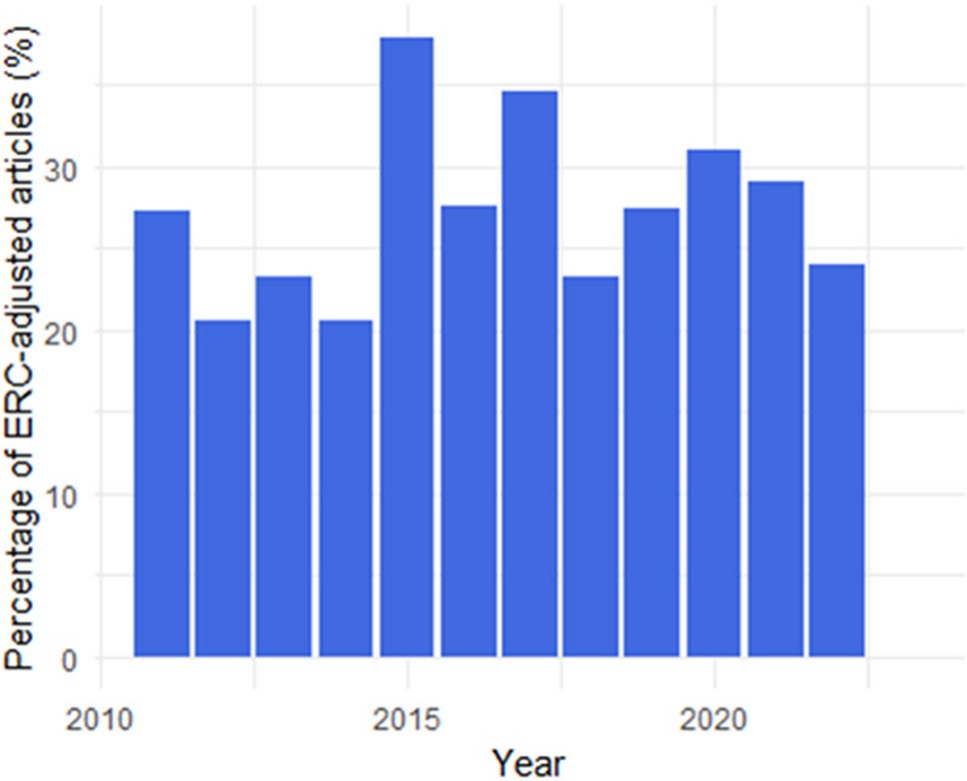
Percentage of studies adjusted for ethno-racial categories(ERCs) per year. Percentage of single-country studies that adjusted for ethno-racial categories out of the total number of studies per year

27% (298/1,085) of one-country studies adjusted for ERCs, with no significant changes during the period 2011–2022 (Cochran-Armitage Trend Test, *p* = 0·53) (Fig. 2). We also did not find differences in the frequency of ERC-confounder adjustment before and after the instigation of the BLM movement in 2013 (chi square *p* = 0·25) or surrounding the increased awareness in health disparities during the COVID-19 pandemic in 2020 (chi square *p* = 0·79).

**Fig. 3:**
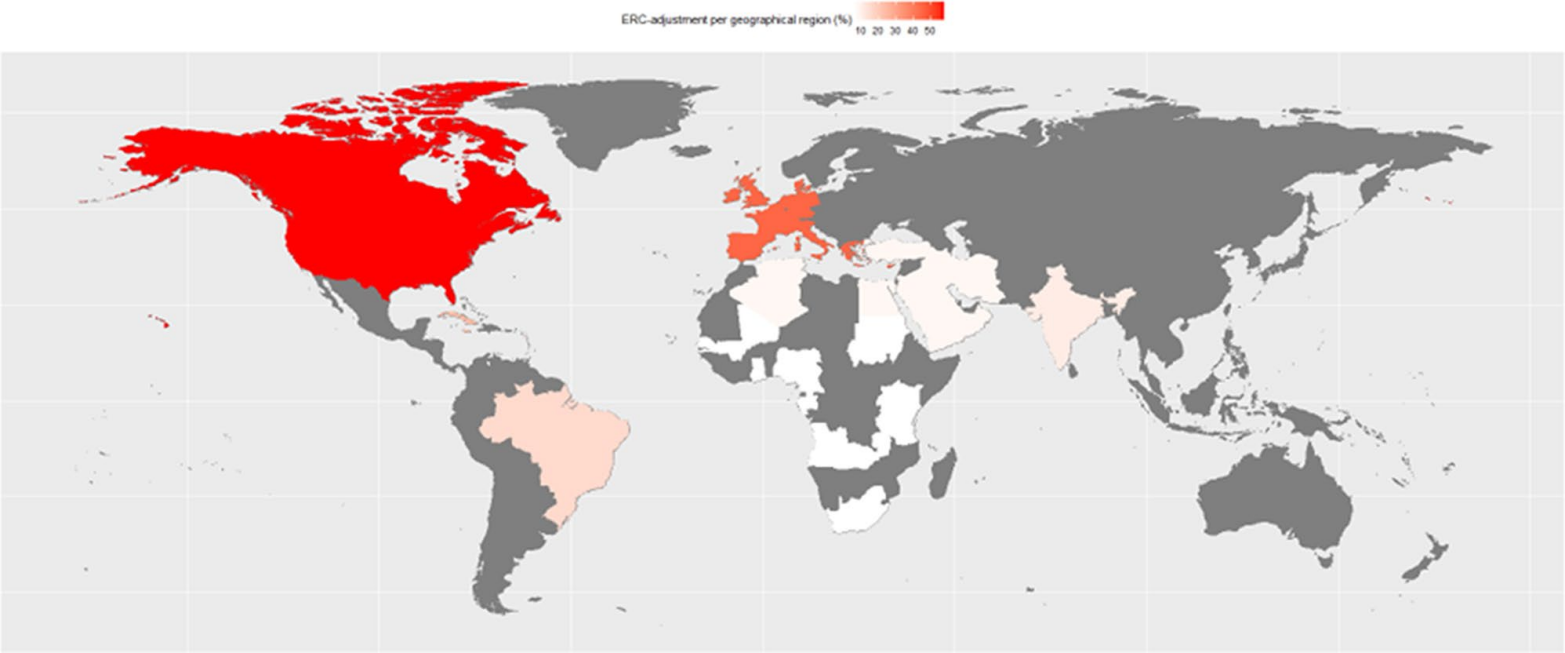
Percentage of studies adjusted for ethno-racial category (ERC) per geographical region. Each country indicates the recruitment country. White represents 0% of studies adjusted for ethno-racial category (ERC), while red represents 100%. Countries shown in grey had no included studies

Studies with a North American study population adjusted for ERCs in 175/302 (57%) of the articles, as well as 45% (58/129) of the studies reporting on a European study population. In contrast, among 23% (7/30) of Caribbean, 11% (5/40) of South Asian, 16% (23/144) of South American, 8% (16/196) of Middle Eastern and North African, and 6% (14/239) of Sub-Saharan African study populations ERC-confounder adjustment was performed (Table 1; Fig. 3). We found a significant association between global region and ERC-confounder adjustment (chi-square test *p* < 0·0001). The odds ratio (OR) for the association between papers coming from a Western country (Europe and North America) and ERC-confounder adjustment was 10·66 (95% confidence interval [CI] 7·75 to 14·66, *p* < 0·0001), compared to non-Western regions (all regions except the defined Western region) indicating a significant association between the global region classified as a Western country and ERC-confounder adjustment.

The Q1 CiteScore was associated with ERC-confounder adjustment. 1,017 of the 1,085 articles were ranked in Scopus with a CiteScore. Out of the 1,017 studies, 451 were published in a Q1 journal. The OR for a paper being published in a Q1 journal was 2·96 (95% CI 2·25–3·90, chi square test *p* < 0·001) for papers performing ERC confounders adjustment compared with those who did not.

### ERC covariate adjustment compared to other covariates

12% of the one-country studies (127/1,085) adjusted for multiple covariates other than demographic variables. This contained covariates such as height, BMI, smoking habit, and parity. Confounder adjustment for these non-demographic variables was less frequent than ERC adjustment (chi-square *p*-value < 0·001).

**Fig. 4:**
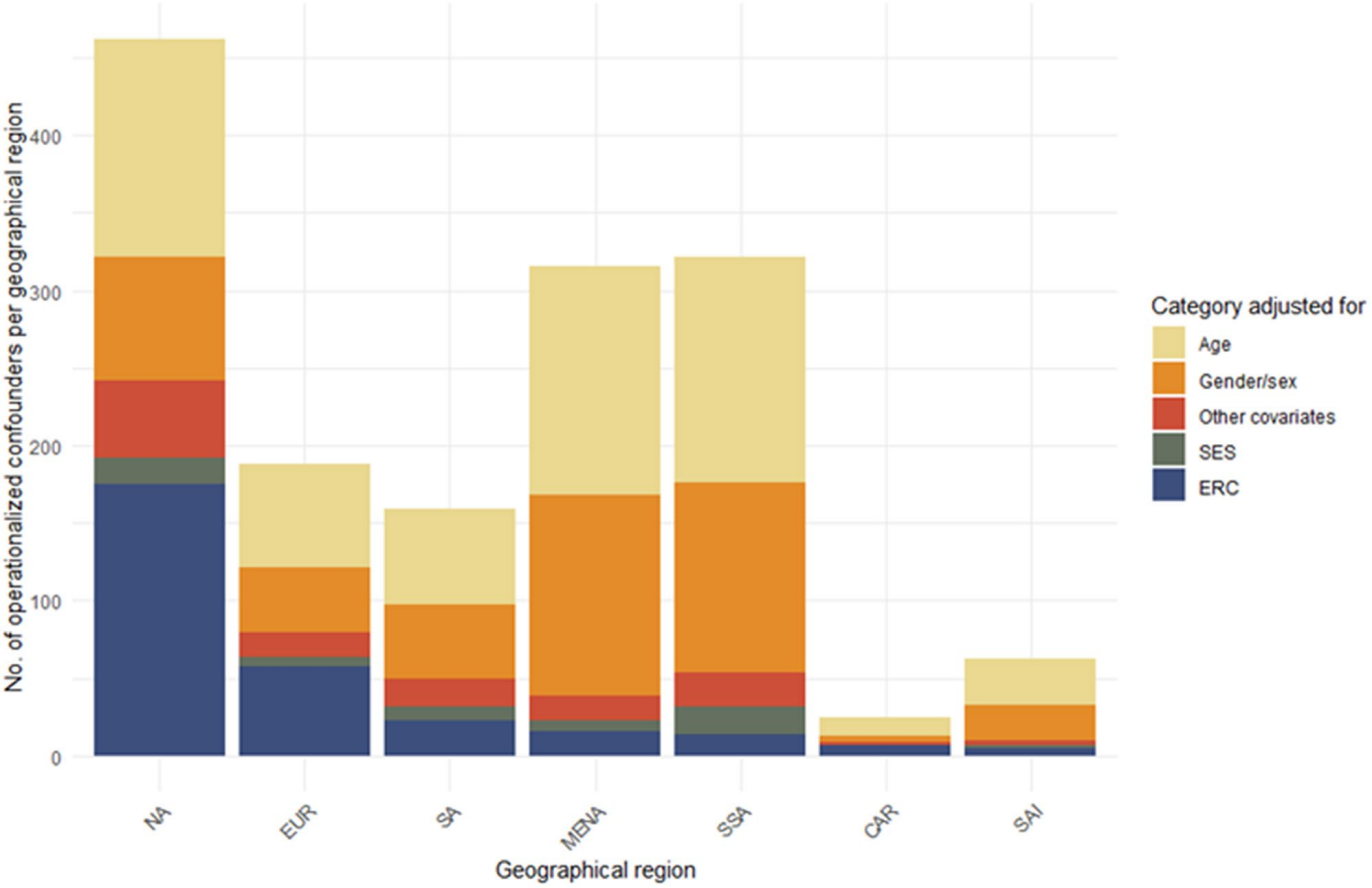
Categories of covariate adjustment across geographical regions Regions refer to recruitment regions. NA = North America, EUR = Europe, SA = South America, MENA = Middle East North Africa, SSA = Sub-Saharan Africa, CAR = Caribbean, SAI = South Asia, SES = Socioeconomic Status, ERC = ethno-racial categories, Other covariates = e.g., height, BMI, smoking, parity. Manuscripts can adjust for multiple covariates; bar lengths do not represent the number of manuscripts per region

We compared ERC-confounder adjustment relative to other demographic confounders: age, gender/sex, and socioeconomic status. Globally, 65% of papers on single-country studies adjusted for age (605/1,085), 41% for gender or sex (446/1,085) and 5% for socioeconomic status (SES) (59/1,085). In Europe, ERCs were the most frequently used characteristic for confounder adjustment and in North America second most frequent, after age-adjustment. In Sub-Saharan Africa, ERC was numerically less frequently used as a confounder than SES (chi square *p* = 0·01) (Fig. 4 and Supplementary Table 4).

Adjusting for SES (OR = 4·32, 95% CI 2·17–8·60, *p* < 0·001) was a significant predictor for ERC-confounder adjustment, whereas adjusting for other variables than before mentioned demographic variables was not significant (OR = 1·55, 95% CI 0·97–2·49, *p* = 0·069). Furthermore, studies with participants originating from North America (OR = 4·42, 95% CI 1·77–11·08, *p* = 0·002) or Europe (OR = 2·56, 95% CI 1·00–6·62, *p* = 0·049), were more likely to adjust for ERCs than study groups from Sub-Saharan Africa (OR = 0·54, 95% CI 0·061–0·49, *p* < 0·001). In this analysis, the Caribbean was used as a reference group. The conditional R-squared of the mixed-effects model was 0·27.

### Contextualization of ERCs

We investigated the contextualization of ERC usage as confounders in the included articles by analyzing their definitions, the rationale for their use, and the context provided for ERCs.

#### Reporting of ERC ascertainment and labeling

In 76% (226/298) of the studies that adjusted for ERCs, the classification criteria were not described. In the papers that did describe their methods (*n* = 72), 61% (44/89) used ERCs from pre-existing databases and 38% (27/72) used self-reported ERCs and 1% (1/72) used a combination of these methods (Supplementary Table 5). Moreover, if ERC-confounder adjustment was performed, 28% (83/298) of the studies did not mention which ERC labels were controlled for. Only a statement that ERC-confounder adjustment occurred, was included. However, the majority of the studies that specified ERCs (69% 206/298) used the label “African”, “Black”, or a derivative.

#### Rationale for ERC adjustment

Of the studies that adjusted for race or ethnicity, 19% (56/298) provided a reason. Of the studies that did not adjust for ERCs, 2% gave an explanation (14/787), for example, the contested background of using race as a proxy for biological differences, or previous literature pointing out that race is not a relevant confounder for their research question. For the specific rationales, extracted from the manuscripts, see Supplementary Tables 6 and 7.

### ERC adjustment within more complicated contexts

We also explored contextualization of ERCs in more complex situations.

#### Cross-national comparative studies

Twenty studies included study populations from several different countries. Two studies were collaborations within Europe, six in Africa, and 13 studies were inter-continental collaborations (Supplementary Table 8). Six of these studies used confounder adjustment. In five publications, the control group was sourced from the same country as the SCD cases. One study used a healthy Congolese reference population as a control cohort while the SCD patients were from France, originating from West or Central Africa, or the West Indies.

#### ERC-adjusted studies with sub-saharan Africa study populations

Of all included geographic regions, studies with Sub- Saharan African study populations were the least likely to adjust for ERCs. Out of the 14 one-country studies with Sub-Saharan African study populations that adjusted for ERCs, five used the label Black, African, or a derivative: “African, Black, African ancestry, Black African, and Indigenous African. (Supplementary box 2) Further refinement into more specific ethnic labels for confounder adjustment, did not occur in any article. Labels such as Yoruba and Igbo in publications with a Nigerian study populations or Akan and Ewe in Ghanaian publications did appear within our search but were only used descriptively. Nine papers did not describe the specific ethnic labels used for confounder adjustment in their studies.

#### Multiracial or multi-ethnic individuals

In 2% (23/1,085) of all papers, a “mixed-race” category was used. However, in none of these papers this category was used as an ethno-racial label to adjust for confounding. In 57% (13/23) of these papers, ERC adjustment on other ERCs was performed. Most of the study groups that did include multiracial or multi-ethnic labels, were sourced from Brazil (52% [12/23]), eight from the United States of America (35% [8/23]), and three from the United Kingdom (13% [3/23]).

**Table 1:**
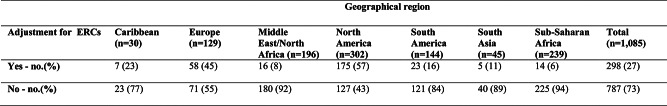
ERC-Confounder adjustment per geographical region (*n* = 1,085)

## Discussion

We found that race and ethnicity were operationalized as confounders in SCD research in nearly one-third of all one-country studies. Studies with a Western study population, were more likely to adjust for ERCs, compared to studies with an African study populations. Describing the method through which race and ethnicity were determined and the rationale for their use, is increasingly being encouraged by medical journals [9, 15, 16, 38, 39]. However, our analysis showed that this methodological practice was scarcely applied. In 76% (226/298) of ERC-adjusted studies, the classification criteria were not described. Our findings are especially relevant in SCD research in Western countries, since patients are more frequently racialized as non-white, compared to the general population [40]. We postulate that authors are aware of demographic differences but are often in doubt on whether and how they should apply these differences in their research. Correspondingly, we found that race and ethnicity are often not replaced by more specific covariates but are instead included in parallel with other potential confounding variables. This approach does not suggest a deliberate effort in deconstructing and replacing ERCs for alternative and possibly more suitable covariates

Since ERCs are highly contextual, it is imperative to provide the context when operationalizing them. Our analysis showed that this was not regarded standard practice. Unfortunately, we did not find an illustrative example in which ERC operationalization was performed in full accordance with current NASEM guidelines. Justification for the use of ERCs as confounders was often not provided. Also, our analysis showed that research articles from Western countries were more likely to correct for ERCs than research articles from non-Western countries. This might be related to historical and/or sociopolitical influences that have shaped biomedical practice [41–43]. For example, the historical impact of colonialism and slavery, as well as the impact of current-day cultural movements such as BLM might be of influence on current methodological approaches. It seems that the use of ERCs as confounders is reinforced in two ways. First, we found that, whenever manuscripts describe a rationale for ERC adjustment, they often referred to related literature which showed a correlation between ERCs and similar study outcomes. Second, studies that perform ERC adjustment are three times more likely to be published in high-impact journals. The decontextualized use of ERCs was also found in reviews examining a variety of general clinical and epidemiological journals [4, 5, 44, 45]. This underlines the prevailing uncertainty with which authors navigate this topic.

In SCD research, the relevance of ERCs is often considered since patients with SCD are often racialized as non-white. Nevertheless, in 72% of all papers, and more specifically in 46% of papers with a Western study population, ERCs were not applied as confounders. Assessing ERCs as a relevant confounder, might be complicated by the fact that individuals of non-European descent are often understudied in biomedical research [46, 47]. This results in a lack of knowledge about the implication of race and ethnicity in SCD outcome measures. SCD researchers also experience ethical challenges. There is a risk of biological and social reification of already minoritized populations, when reporting and operationalizing ethnic and racial background. ERCs identified as confounders, are often converted into race correction factors and applied in clinical algorithms [7, 48, 49]. Even if the use of ERCs in research is performed with context, this danger is always present.

Covariates that are responsible for variation in health outcomes are also dependent on the context. Biomedical researchers should therefore engage with social scientists when designing and reporting research.

Confounders, by definition, may distort study outcomes. However, the current, decontextualized use of ERCs in SCD, obscures the specific confounding pathway. Furthermore, this often leads to reinforcing ERCs as biological labels, such as using them as a proxy for genetic ancestry. In 2022, NASEM issued guidelines saying that race and ethnicity are inadequate proxies for human genetic variability. Researchers should try to pinpoint the specific information relevant to their research questions [50]. When studying health disparities and trying to control for genetics, NASEM recommends using the term “genetic similarity” instead of an ER label [50]. Genetic ancestry is defined as the population origin of a person’ alleles at polymorphic sites and can be estimated against a global reference of diverse individuals [51]. However, in the absence of genetic data, race and ethnicity are very poor proxies of genetic ancestry. Without relevant genetic information, there is an increased risk of defaulting to racial categories [52, 53]. ERCs have been shown to be particularly inadequate for recently admixed populations, such as those as labelled “Hispanic or Latino” by the US census [3]. 

In absence of more granular data, such as genetic data to support one’s assumptions, it becomes more crucial to consult additional sources that support population-level differences, relevant to study outcomes and exposures, such as documented population differences in reference lab values [54]. If this data is not available, researchers should perform literature reviews, sensitivity analyses, or propose well-supported hypotheses for population differences. Secondly, race and ethnicity often intersect significantly with environmental variables. Prior knowledge of how the study population is being affected by these variables (e.g. interpersonal racism, resources-deprived neighborhoods and air quality, guides researchers towards collecting relevant data. Standardization and harmonization are nearly impossible when relying on race and ethnicity, globally, but become more feasible with the implementation of granular measures and the availability of validated methods. The PhenX toolkit offers standard data collection protocols, including questionnaires on perceived discrimination and air quality data extraction [55]. The Neighborhood Atlas provides open data on neighborhood disadvantage in the United States, which can be used in models to study systemic racism [41]. Using validated means of data collection as well as population data itself can be combined with more sophisticated models that examine ethno-racial health disparities through the lens of systemic racism, as have been proposed in the literature [56–58]. It is essential to advance the field of research by moving beyond the limitations of ethnic or racial categorizations and focus on the underlying determinants driving health disparities.

For the operationalization of ERC in multiracial or multiethnic participants, it may be relevant to apply different categorization schemes when comparing results in one study. The way in which multiracial participants are processed in the study can change outcome estimates for other ERCs identified as well. In cases with multiracial populations, it is important to try to hypothesize the mechanism that might drive the outcome of interest and how multiracial identity in a particular study population might tie into it [17]. In this context, NASEM has proposed new methods for categorizing multiracial identity in biomedical research [17].

This analysis of the operationalization of ERCs as confounders in a racialized disease as SCD, is a novel contribution to the existing body of literature on the application of ERCs in biomedical research. Previous research on this topic, mainly focused on high-income countries or publications in high-impact journals [13]. By examining methodological practices in SCD research, we were able to analyze this from a broader perspective.

The challenge with operationalizing race, ethnicity, or similar factors such as genetic similarity lies fundamentally in the act of categorization itself. In a research environment with limited resources, there is always a trade-off between quality, financial cost, and time investment. Especially when studying study participants of color, which is the case in SCD research, this challenge is unlikely to be resolved. Even if genetic ancestry data were available and accessible for research participants, there may still be an albeit small residual component reflecting other biological-biological and biological-environment interactions, such as metabolomics, epigenetics, and the microbiome. While the scientific method may never achieve perfection, it must strive to be responsible. If ERCs are considered relevant, providing context should be a requirement.

In conclusion, the heterogeneity in the use of ERCs in SCD research has been shown in this review. These findings might have consequences for ERC-confounder adjustment in biomedical research in general. It is of the utmost importance to consider a more precise variable which is better suited to the research question, before using ERCs.

By redirecting the focus toward researching more specific, qualitatively better health determinants, we depart from the troubling trend of continuously relying on race and ethnicity. In this way, biomedicine mitigates the unintended perpetuation of health disparities and draws closer to contributing to outcomes in a more equitable way.

## Electronic supplementary material

Below is the link to the electronic supplementary material.

Supplementary Material 1

## Data Availability

Our data is now accessible on GitHub under the GNU General Public License version 3.0 (GPL 3.0), along with the analyses we’ve conducted. This license permits users to freely access, modify, and distribute both the data and analyses. However, any modifications or derivative works must also be released under the GPL 3.0 license. This framework encourages collaboration and transparency while upholding licensing terms. Feel free to explore and utilize our GitHub data repository.URL: https://github.com/AidaasinAyda/SCD_conf_correction.git.
