# Supplementary material for: The use of race and ethnicity in sickle cell disease research
Source: BMC Med Res Methodol. 2025 Mar 7;25:63. doi: 10.1186/s12874-025-02513-5 (PMC11887362; doi:10.1186/s12874-025-02513-5)

Appendix tables and figures

The use of ethnicity and race in sickle cell disease research

**Table of contents**

| **Figures** | **Page** |
| --- | --- |
| **Figure 1**. **PRISMA ™ flow diagram of the selection process for the quantitative and qualitative literature analysis of SCD research.** | 2 |
| **Figure 2.** **Percentage of** **studies adjusted for ethno-racial categories(ERCs) per year** | 3 |
| **Figure 3. Percentage of studies adjusted for ethno-racial category (ERC) per geographical region.** | 4 |
| **Figure 4. Categories of covariate adjustment across geographical regions** | 5 |
| **Supplementary Figure 1. HbS allele frequency (AF) and no. of published manuscripts per recruitment country.** | 22 |

| **Tables** | **Page** |
| --- | --- |
| Supplementary table 1a. Used Embase search terms | 8 |
| Supplementary table 1b. Used Medline search terms | 8 |
| Supplementary table 2. Countries assigned to geographical region | 9 |
| Supplementary table 3. Number of single-country studies per country | 10 |
| Supplementary table 4. Adjustment of demographic and other variables per region | 12 |
| Supplementary table 5. Methods used to determine ERCs | 14 |
| Supplementary table 6. Rationale for ERC-adjustment specified | 15 |
| Supplementary table 7. Rationale for not performing ERC-adjustment specified | 19 |
| Supplementary table 8. Countries of cross-national comparative studies | 15 |

| **Boxes** | **Page** |
| --- | --- |
| Box 1. Population labels and geographical regions | 6 |
| Supplementary Box 2. Derivatives of African or Black descent | 21 |

**Figure 1**. **PRISMA ™ flow diagram of the selection process for the quantitative and qualitative literature analysis of SCD research.**

**
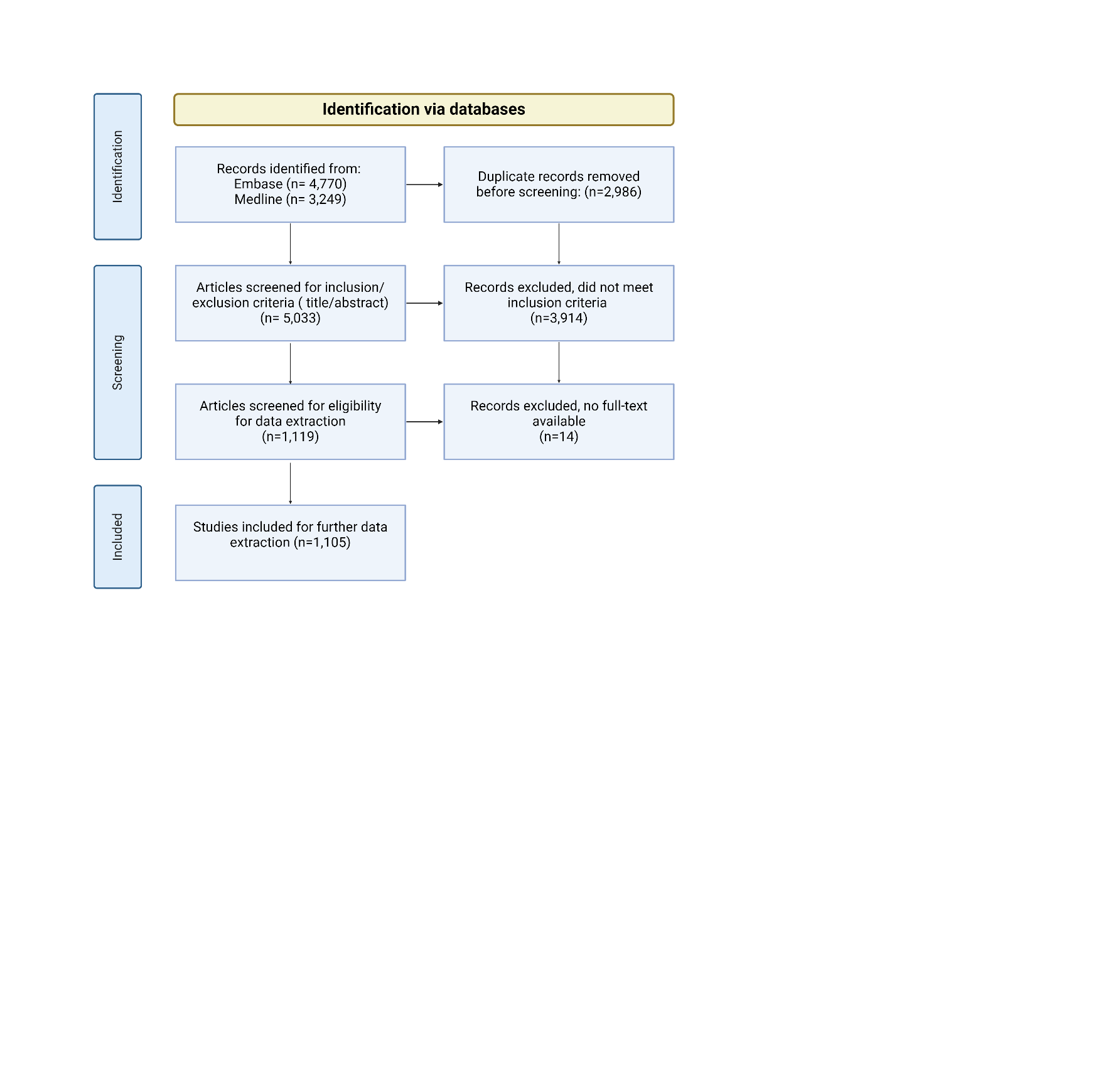
**

**Figure 2.** **Percentage of** **studies adjusted for ethno-racial categories(ERCs) per year**


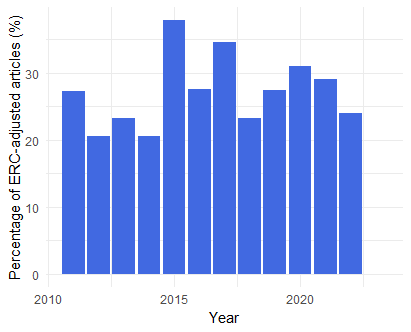


**Figure 3. Percentage of studies adjusted for ethno-racial category (ERC) per geographical region.**


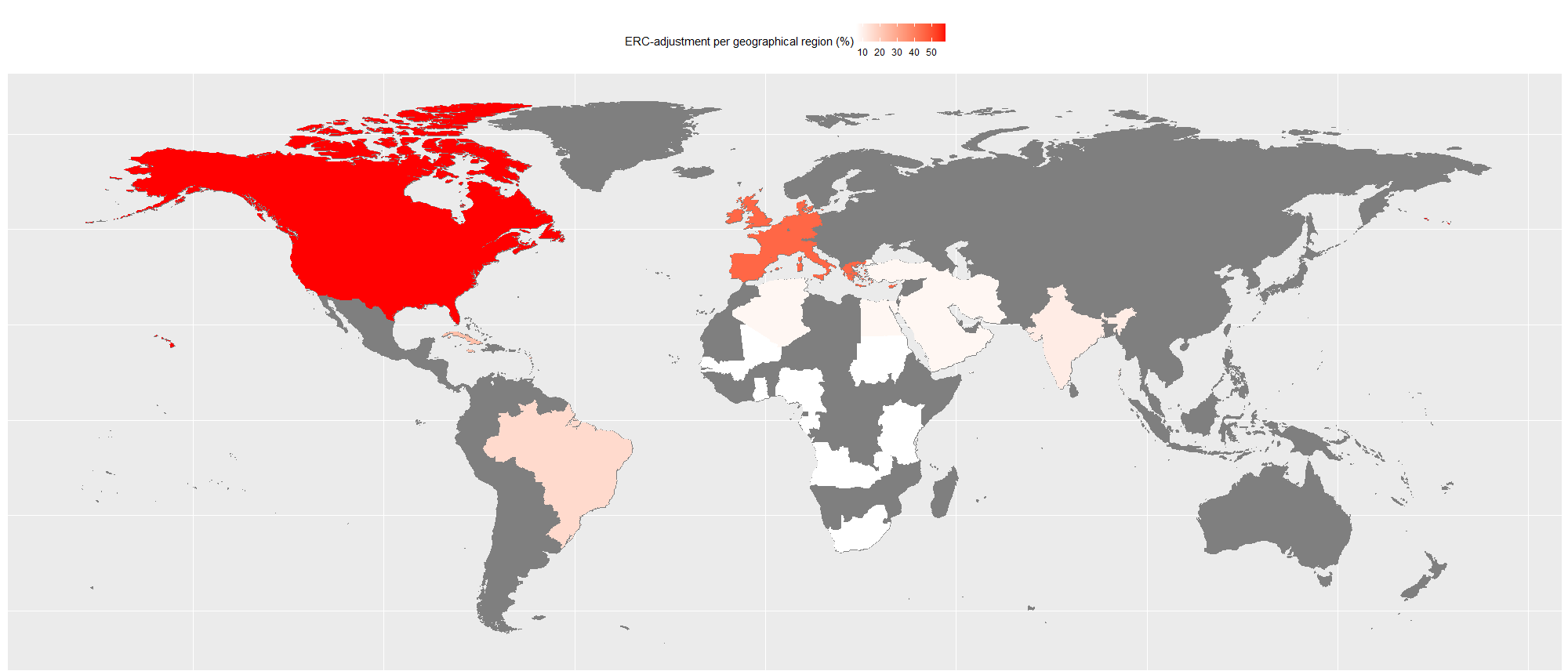


**Figure 4. Categories of covariate adjustment across geographical regions**


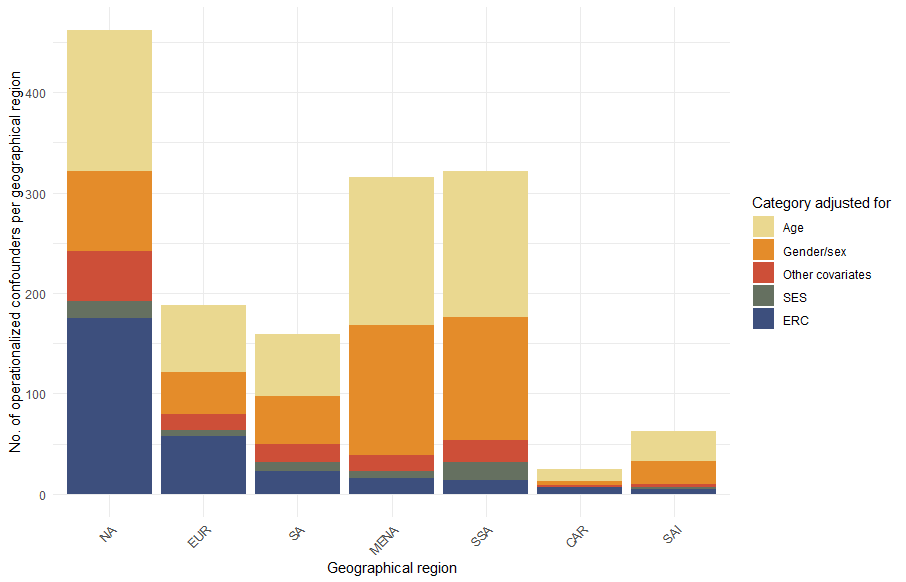


**Box 1. Population labels and geographical regions**

Ethno racial categories (ERC): We did not distinguish between ethnicity and race, considering that in practice the definitions have major overlap.

Western: We use “Western countries” for primarily countries in Europe and North America. This term is used as an overall term to refer to high-resource countries.

“Racialized as {insert ethno-racial label}”: We prefer using the term “racialized as” to draw emphasis to the fact that racial labels are often assigned to individuals, even though they would not necessarily identify with these labels themselves. Racialization could be a precursor to racial discrimination. By using this language, we aim to avoid assigning a fixed racial identity to the individuals discussed, while still acknowledging the impact of racialization, marginalization, and discrimination.

Supplementary material

**Supplementary table 1a.** **Used** **Embase search terms**

| Search terms | |
| --- | --- |
| Sickle cell Disease | ('sickle cell anemia'/exp/mj OR (sickle-cell*):ti) |
| Study designs | AND ('controlled study'/exp OR 'control group'/de OR 'cohort analysis'/de OR 'longitudinal study'/de OR 'prospective study'/de OR 'retrospective study'/de OR ((controlled NEAR/3 (stud* OR trial*)) OR case-control* OR control-group* OR (match* NEAR/3 control*) OR cohort* OR longitudinal* OR prospective* OR retrospective*):Ab,ti) |
| Filters | NOT ([animals]/lim NOT [humans]/lim) AND [2011-2030]/py NOT [conference abstract]/lim AND [english]/lim |

**Supplementary table 1b**. **Used** **Medline search terms**

| Search terms | |
| --- | --- |
| Sickle cell Disease | (exp *Anemia, Sickle Cell/ OR (sickle-cell*).ti.) |
| Study designs | AND (Case-Control Studies/ OR Controlled Before-After Studies/ OR exp Cohort Studies/ OR ((controlled ADJ3 (stud* OR trial*)) OR case-control* OR control-group* OR (match* ADJ3 control*) OR cohort* OR longitudinal* OR prospective* OR retrospective*).ab,ti.) |
| Filters | NOT (news OR congres* OR abstract* OR book* OR chapter* OR dissertation abstract*).pt. AND english.lg. |

**Supplementary table 2**. **Global regions and corresponding countries**

| Global region | Countries of origin from included studies |
| --- | --- |
| Middle-Eastern North African (MENA) | Jordan, Turkey, Egypt, Saudi Arabia, Kuwait, Kurdistan, Bahrain, Iraq, Tunisia, Palestine, Iran, Oman, Yemen, Algeria |
| South Asian (SAI) | India |
| South American (SA) | Brazil, French Guyana |
| European (E) | UK, France, the Netherlands, Switzerland, Germany, Denmark, UK, Ireland, Belgium, Cyprus, Greece, Italy, Portugal, Spain |
| Sub Saharan African (SSA) | Nigeria, Senegal, Mali, Ghana, South Africa, Uganda, Cameroon, Tanzania, Republic of the Congo, Gabon, Ivory Coast, Sudan, Kenya, Togo, Angola, Democratic Republic of the Congo, |
| North American (NA) | Canada, USA |
| Caribbean (CA) | Jamaica, Guadeloupe, Martinique, Cuba |

**Supplementary table 3.** Number of manuscripts per country

| Country | Count |
| --- | --- |
| USA | 290 |
| Nigeria | 156 |
| Brazil | 144 |
| Egypt | 68 |
| Turkey | 47 |
| India | 45 |
| UK | 38 |
| France | 34 |
| Saudi Arabia | 33 |
| Ghana | 23 |
| Jamaica | 15 |
| Netherlands | 15 |
| Guadeloupe | 14 |
| Tanzania | 13 |
| Canada | 12 |
| Greece | 12 |
| Sudan | 11 |
| Italy | 10 |
| Kuwait | 10 |
| Cameroon | 9 |
| DRC | 9 |
| Iran | 8 |
| Iraq | 7 |
| Bahrain | 6 |
| Belgium | 6 |
| Oman | 6 |
| Tunisia | 5 |
| Germany | 4 |
| Uganda | 4 |
| Ireland | 3 |
| Kenya | 3 |
| Angola, Ivory Coast, Palestine, Portugal, Senegal, Switzerland | 2 |
| Algeria, Cuba, Cyprus, Denmark, Gabon, Jordan, Kurdistan, Mali, Republic of the Congo, South Africa, Spain, Yemen, Zambia | 1 |

**Supplementary table 4.** Adjustment of demographic and other variables per region

| Geographic Region | Demographic Category | Articles using adjustment, % (n) |
| --- | --- | --- |
| Caribbean (n=30) | Age | 40 (12) |
|  | ERC | 23 (7) |
|  | Gender/Sex | 13 (4) |
|  | Other | 7 (2) |
|  | SES | 0 (0) |
| Europe (n=129) | Age | 52 (67) |
|  | ERC | 45 (58) |
|  | Gender/Sex | 32 (41) |
|  | Other | 12 (16) |
|  | SES | 5 (6) |
| Middle-East North Africa (n=196) | Age | 76 (148) |
|  | ERC | 8 (16) |
|  | Gender/Sex | 66 (129) |
|  | Other | 8 (16) |
|  | SES | 4 (7) |
| North America (n=302) | Age | 46 (140) |
|  | ERC | 58 (175) |
|  | Gender/Sex | 26 (80) |
|  | Other | 17 (50) |
|  | SES | 6 (17) |
| South America (n=144) | Age | 43 (62) |
|  | ERC | 16 (23) |
|  | Gender/Sex | 33 (47) |
|  | Other | 12 (18) |
|  | SES | 6 (9) |
| South Asia (n=45) | Age | 67 (30) |
|  | ERC | 5 (11) |
|  | Gender/Sex | 51 (23) |
|  | Other | 6 (3) |
|  | SES | 4 (2) |
| Sub Saharan Africa (n=239) | Age | 61 (146) |
|  | ERC | 6 (14) |
|  | Gender/Sex | 51 (122) |
|  | Other | 9 (22) |
|  | SES | 8 (18) |

ERC = Ethno-racial category; SES = Socioeconomic Status

**Supplementary table 5. Methods to determine ERCs**

| Method | No. |
| --- | --- |
| Pre-existing database registration | 44 |
| Several ways | 1 |
| Self-report | 27 |
| Total | 72 |

**Supplementary table 6.** Rationale for ERC-adjustment specified

| Record number | Rationale |
| --- | --- |
| 138 | In addition, it is important to acknowledge that FAZ area can vary with ethnicities. An enlarged FAZ area is not specific to patients with SCD but is also a physiological variation for unaffected subjects from African origin. |
| 144 | Although previously published studies report mixed results regarding the independent effect of race on COVID-19–related outcomes, it is undebatable that Black persons in the United States have been disproportionally affected by this evolving pandemic. |
| 166 | Model 2 only included those identified as Black or AA in the controls, because sickle cell disease is strongly associated with ancestry, and race is associated with mortality in patients with kidney failure. |
| 181 | Our results cannot be explained by racial differences because we included race-matched participants in the control group. This inclusion is important because patients of African descent have thinner central macular thickness and nerve fiber layers. |
| 207 | Previous research has described a lower incidence of acute appendicitis in black or African American patients compared to other racial groups. Buckius et al reported that blacks or African Americans have an approximately 50% lower incidence of acute appendicitis compared to whites (in a comparison of groups composed of all ages), and Anderson et al reported higher rates of appendicitis in both whites and Hispanics, and lower rates in blacks or African Americans and Asians. Blacks or African Americans comprised 75.1% of our SCD cohort, and only 33.5% of our control cohort, although the number of blacks or African American patients in the control cohort may be underestimated since a majority (54.5%) of the control cohort reported their race as either “Multiracial” (24.0%) or “Other/Unknown/Declined to answer” (30.5%). |
| 229 | In a study using HCUP data, Tangel et al. found that black women were more likely than white women to die in-hospital and have a longer expected LOS, on average, across most insurance categories and quartiles of median income. As a result of this potential confounder, we performed sensitivity analyses of each of our multivariable models in a sample of black patients only, who comprised 37% of our SCD population. |
| 382 | African Americans with cancer experience poor pain control and decreased perception of control over pain as well as higher death rates compared with all other racial or ethnic group (…) Anecdotes from patients with SCD indicate that pain is inadequately managed because healthcare providers lack empathy for their pain condition, which they view as less painful than cancer pain. |
| 569 | It has been shown that there is substantial interethnic diversity in the distribution of eNOS variants. Brazil has a highly heterogeneous population with an ethnic composition that varies from region to region. |
| 731 | Because SCD is most common among the black population, this analysis was repeated using only black patients to eliminate a possible confounder. Previous literature has shown that health care disparities exist and have been linked to social determinants of health such as race, health insurance primary payer status, and wealth and wealth distribution |
| 1051 | The absence of updated local reference data for spirometry values for Nigerians compelled comparison with a control group who are ethnically similar and likely to have similar nutritional and environmental exposures that may influence lung function. |
| 1130 | CD patient-derived (Martins et al., 2018) and age- and sex-matched control iPS cell lines from different ethnic origins provide useful tools for studies aiming at addressing the influence of genetic diversity in the disease pathogenesis, drug discovery and gene editing. |
| 1174 | Since our study was focused on sickle cell disease and its associated vasculopathy, the ethnic makeup of our  control group was similar to our patient population, 80–90% African-American and 10–20% Hispanic. It is known that nitric oxide dependent vascular function measures in conduit arteries (FMD) and the microcirculation (resting perfusion and PORH) are decreased in African-American subjects without sickle cell disease. |
| 1181 | While published studies have established normal standards based on age and sex, African Americans have been shown to have reduced tolerance to experimentally-induced pain. |
| 1236 | African Americans share a disproportionately high burden of diabetes and diabetes-related complications, with an up to two-fold increased likelihood of developing diabetes compared to non-Hispanic White adults. These racial disparities in diabetes may be  attributable to lifestyle differences as well as socioeconomic and genetic differences between  these populations. |
| 1244 | An additional stratified model was run for patients of black race only, as they are more likely to inherit SCD and historically have been shown to have poorer surgical outcomes. |
| 1287 | It is important to consider other variables that are known to affect anti-Müllerian hormone (AMH) such as ethnicity, smoking, body mass index (BMI), and usage of combined oral contraception. |
| 1319 | Seventy-eight consecutive non-SCD control patients were selected, matching for age, gender, and race as closely as possible to minimize any potential bias. Because the demographics of several SCD patient scans were listed as both “Black/Hispanic,” a control was considered a race/ethnicity match if the demographics was considered a race/ethnicity match if the demographics were listed as either “Black” or “Hispanic” when the age and gender were also a match. |
| 1525 | Group differences carry more weight as they are matched for age, ethnicity, gender, and SES, reducing the likelihood of spurious effects. This stringent approach allows us to elucidate differences due to disease without the influence of previously reported confounding factors. |
| 1531 | The degree of RH genetic diversity differs depending on ethnic group, with increased diversity in African populations, and hence patients with sickle cell disease (SCD) compared with Europeans who represent the majority of blood donors. Approximately 85% of African American patients with SCD carry at least 1 RH allele that differs from that commonly found in white blood donors. |
| 1548 | We matched for these variables because SCD-related bowel ischemia occurs at a younger age than the general population, SCD is more prevalent in blacks, and ischemic bowel disease occurs more in women |
| 1811 | The majority of individuals with SCD are black and findings from a nationally representative sample of healthy youth using time-diaries found that black adolescents report sleeping less on weekdays and weekends compared with white, Hispanic, and Asian adolescents. |
| 1872 | For percent predicted TLC, a 12% adjustment was used to account for the effect of African American race |
| 1874 | We selected the patients’ healthy HbAA siblings as controls in order to minimise the potential effect of dietary background and genetic factors on the variables under investigation. |
| 1921 | Healthy ethnically-matched controls were recruited from the hospital staff. Groups were age (10 years range) and ethnicity matched since there are racial differences in coagulation parameters. |
| 1927 | African Americans also experience greater pain sensitivity and reduced CPM than Europeans, suggesting that our small pilot study might have been underpowered. |
| 1976 | Children were well matched to limit ethnical, gender and age influences on skin blood flow |
| 2033 | It has been demonstrated that skin pigmentation may interfere at some extent with the quality of NIRS signal in attenuating light reflectance in dark skins [31]. All children were Afro-Caribbean and had homogeneous skin pigmentation: they were classified by the Fitzpatrick scale between V and VI (on a scale ranging from I to VI). Indeed, the differences observed between the three groups  were not related to skin color differences |
| 2293 | Women were matched by age at delivery, parity and ethnicity to control for birth weight variations pertaining to ethnicity and parity. Babies born to black mothers have been shown to be more likely to be of LBW when compared to babies born to white mother |
| 2298 | Race-matched controls are imperative in pain studies as data support race and ethnic differences in pain sensitivity with African American people displaying increased pain sensitivity. |
| 2356 | All the main analysis was confined to patients in the SCD and control cohorts with a HES record that specified that they were Black. We did this knowing that HES data on ethnicity is incomplete; but we wanted, as best as we could, to distinguish associations attributable to SCD from those that may be attributable to ethnicity. |
| 2383 | Results were expressed as percent predicted for height, age, and sex using the ethnic-specific reference equations for spirometry and the European Community for Steel and Coal Statement of the European Respiratory Society reference equations for lung volumes and gas transfer for patients over 18 years of age and those of Rosenthal et al. for children under eighteen. |
| 2477 | Conditioned pain modulation is believed to reflect endogenous opioid function, and African Americans show reduced CPM responses when compared to non-Hispanic white participants. |
| 2516 | All of the SCD children and the controls were African or Caribbean, thereby avoiding confounding from ethnic differences in exhaled NO. It should also be noted that the sample size was relatively small in this study, which may have limited our ability to detect subtle differences between the SCD and control groups |
| 2540 | Previous studies have reported higher rates of PE in the black population compared to other ethnic groups, and, in particular, have pointed to a higher rate of provoked PE |
| 2556 | Furthermore, determining thresholds specifically for patients with SCD with proper race-matched controls is important as pain sensitivity is shown to vary based on ethnicity with African American people have increased sensitivity to pain as assessed by QST measures. |
| 2570 | The aim of the study is to establish the MBL genotypes in the SCD patients as well as in the Omani ethnic population. |
| 2846 | Chlamydia rates are disproportionately higher among older teenagers, young adults, and African American patients compared with the national average. |
| 2903 | Other studies have reported racial differences in foveal pit morphology between Caucasians and black people, indicating that the latter have significantly deeper and broader foveal pits than the former. |
| 2907 | Mortality rates were calculated by SCD type and by selected demographic and clinical risk factors, including the child’s sex, birth weight, and gestational age, as well as maternal age, race/ethnicity, country of birth, and residence at delivery. |
| 3064 | There are many factors involved in modulating pain and sensory processing through facilitation or inhibition. Increased pain unpleasantness, but no difference in reported pain intensity, has been described using suprathreshold painful stimulation in African American adults compared with Caucasian adults. |
| 3155 | VC was less than the lower limit of normal, identified as a z-score <−1.64 based on the ethnic specific reference range of Quanjer et al. |
| 3177 | We may have overestimated the prevalence of atopy in control and SCD children by using an arbitrary cut-off value for total serum IgE. Total IgE increases with age and there are ethnic differences, such that total IgE is approximately 1.3 times higher in Afro-Caribbean compared with white British children. |
| 3219 | The adverse effects of high neutrophil count and AM expression on the severity of SCD could have contributed to the lower reference range of blood neutrophil count observed in people of black African ancestry relative to other racial groups. |
| 3296 | Our study reveals that the mean age of menarche of 13.1 years normal/controls was delayed by 4.0 years in SCD females as observed in Jamaican SCD girls, which indicates that ethnic variation may be associated with menarche. |
| 3299 | However, in view of lack of sufficient studies in different ethnic population of India and other countries on eNOS gene polymorphism in SCD patients, further studies with increased number of individuals carrying these polymorphisms may be planned to extend the results of this study. |
| 3342 | Moreover, the higher frequency of MTHFR and FVL mutants in our studied population may also be due to study design, different ethnic selection, interaction with other genetic polymorphisms/or environmental factors within and among ethnic groups. |
| 3506 | In the African Descent and Glaucoma Evaluation study, patients of African descent had thicker peripapillary RNFLs than those of European descent. |
| 3532 | Race-matched controls were vital since data indicate African Americans have lower tolerance to experimentally induced pain and increased sensitivity to thermal and mechanical testing. |
| 3727 | Because all SCD participants were African American, we recruited only African American control participants. |
| 3984 | Moreover, studies establishing normative data in healthy control patients have demonstrated that, in terms of central subfield (CSF) thickness, the central macula is significantly less thick in African Americans and female subjects than in Caucasians and males using the Stratus OCT9 and Spectralis spectral-domain OCT (SDOCT). |
| 4133 | We restricted this sample to those who reported their primary race as Black since the majority of individuals with SCD are Black and without race/ethnicity information from the Truven database, we were unable to match more granularly. |
| 4148 | Chronic graft-versus-host disease has also been attributed to higher risk of death in African Americans after transplantation for aplastic anemia. |
| 4178 | Given that SCD is associated with Black race and that race represents a social determinant of health that can impact rates of cancer treatment, we compared Black versus the remaining combined non-Black racial categories. |
| 4212 | Significant racial disparities exist in the morbidity and mortality from heart failure (HF). It has been established that HF occurs more in AA at an earlier age and with far worse morbidity, prognosis, and mortality than Caucasians or other ethnicities. |
| 4279 | In addition to managing an inherited chronic medical condition, those living with SCD are also confronted with the race-related stress and social disadvantage that come with being a Black person in America. To examine the racial, ethnic, and cultural influences on mental disorders and mental health. |
| 4318 | Our results also reflect a variation amongst races as our data showed no significant change when comparing children with sickle cell disease to healthy black children of a similar age, but did show a significant difference amongst our participants in contrast to their white cohorts in the aforementioned study. |

**Supplementary table 7.** Rationale for not performing ERC-adjustment specified

| Record number | What rationale |
| --- | --- |
| 9 | Because ethnicity is associated with both carrier status and health services use, it was an important potentially confounding factor in this study. However, ethnicity is difficult to define and cannot be reliably ascertained using data available. |
| 885 | First, the sample size was insufficient for the assessment stratified by gender, age, race and BMI and pre-cluded the subanalysis according to the classification of ventilatory patterns. Also, a possible sample bias can be suggested in relation to ethnic differences. |
| 895 | Second, the healthy controls are made up of all different ethnic backgrounds, while all of the SCD patients are African American. It is unknown if there are true differences in atrial strain measurements among different ethnic groups. |
| 1461 | No echocardiographic differences were found between race or ethnicity, so all controls were combined into a single group. |
| 1621 | Ideally, we would have liked to compare IPD rates in children with SCD with IPD rates in Afro-Caribbean children without SCD, and with children with and without comorbidities. However, ethnicity is poorly reported and we do not have reliable national denominator data for children with different comorbidities. |
| 1648 | The sample size was small and lack of information constitutes a shortcoming of the study. Therefore, future studies with large sample size are needed in different ethnic groups with carefully matched cases and controls to confirm the results of our TNF and  the -251A>T IL-8 polymorphism and other gene polymorphisms as a disease susceptibility factor in SCD. |
| 2157 | Subjects and controls were not matched by age, gender or race. Prior studies report that age is an important factor in the prevalence of PFO but sex and race are not. We re-evaluated this by risk adjusting the influence of demographic features (group, sex, race, and age at echo) on the prevalence of potential shunting or pulmonary shunting using logistic regression models. |
| 2368 | One limitation of the study was that the control group was not matched for ethnicity and socioeconomic status as the controls were recruited from the community. However, the association analysis demonstrated that ethnicity and socioeconomic status did not confound the effect of disease in our study since significant association was observed only in the SCD group. |
| 3019 | Nevertheless, comparison across populations from different ethnicities and geographical location needs some caution, as the advantageous effect of α-thalassemia is clearly associated with altitude, age of individuals and endemicity of malaria. |
| 3580 | All patients with SCD in our study were self-identified African American. From a genetic standpoint, this is a term that is not rigorously defined, and as a group our patients had varying degrees of genetic admixture, both of Caucasian and of African origin. |
| 3802 | To rule out the fact that our results could be influenced by the race, we performed additional thrombin generation tests in 60 volunteers Sub-Saharan Africans (15 women and 15 men) and age matched Caucasians (15 women and 15 men). |
| 4230 | There was no racial matching between these patients and the normal controls because of the lack of sufficient normal cases with CTA. |
| 4253 | Future research which specifically recruits people from underrepresented racial and ethnic groups and applies optimal methods to produce fully adjusted scores accounting for race, ethnicity, education, and other social factors is important for building on these exploratory findings. |
| 4306 | Notably, race and ethnicity were not evaluated in adolescents with SCD as all participants were Black and non-Hispanic. However, in our sample of Black adolescents without SCD (n = 15), their mean NVS score was 2.27, more similar to our adolescents with SCD than those without. Thus, in congruence with previous research, race may play a significant role in adolescent health literacy. |

**Supplementary table 8. Countries of cross-national comparative studies**

| **Record number** | **Country 1** | **Country 2** | **Country 3** |
| --- | --- | --- | --- |
| 163 | Canada | Switzerland | - |
| 192 | Switzerland | France | Germany |
| 197 | Mali | Senegal | - |
| 222 | Denmark | Australia | UK |
| 508 | Senegal | Brazil | France |
| 786 | Martinique | Guadeloupe | French Guiana |
| 975 | Nigeria | UK | - |
| 997 | Mali | USA | - |
| 1148 | Brazil | Ireland | - |
| 1383 | Mali | Senegal | Cameroon |
| 1529 | Mali | Togo | - |
| 2300 | Cameroon | Ivory Coast | Gabon |
| 2302 | France | Mali | - |
| 2419 | France | Guadeloupe | - |
| 2463 | Kenya | Tanzania | - |
| 2873 | Mali | USA | - |
| 2881 | USA | Mali | - |
| 3288 | Nigeria | Jamaica | Ghana |
| 3822 | Jamaica | UK | - |
| 4058 | France | DRC | - |
| 4187 | DRC | Ivory Coast | Senegal |

**Supplementary Box 2.** Derivatives of African or Black descent used by studies that adjusted for ERCs

African, African American, Black ancestry, African Caribbean, Black, Black or African American, African descent, Blacks or African Americans, Black and African American, non-Hispanic Black, Black British, African american, Sub Saharan African or Caribbean, African ancestry, Black or African descent, African American non Hispanic, African American or Caribbean African, Afro Brazilian, Afro Caribbean, Western African and Caribbean, Black or Hispanic, African American/ Black Non-Hispanic, African or Caribbean ethnicity, African American or Black, Non-Hispanic Black, Black or African or African American, Black or Afro-Caribbean, Sub- Saharan african ethnic origin, Afro-Caribbean, African Guadeloupians, Black British or Black African or Black Other, Black or African or any other Black background, African Brazilian, African Caribbean or West African descent, African American or Caribbean origin, African or Caribbean ethnic origin, African or African Caribbean, African Caribbean or African ethnic origin, African Carribean or African Ethnic, Afro-descendants Brazilians, African Americans, African descendants, African Ancestry, Black African or Black British, African ethnicity, African American descent, African origin, Black African Ancestry, Afro-Brazilian, Black African children, African background, Indigenous Africans, African-American, Black either African American or Black Caribbean, Black: African American or Caribbean Black, Black and African Ancestry markers, Black ethnicity

ERC = Ethno-racial category

**Supplementary Figure 1. HbS allele frequency (AF) and no. of published manuscripts per recruitment country.**


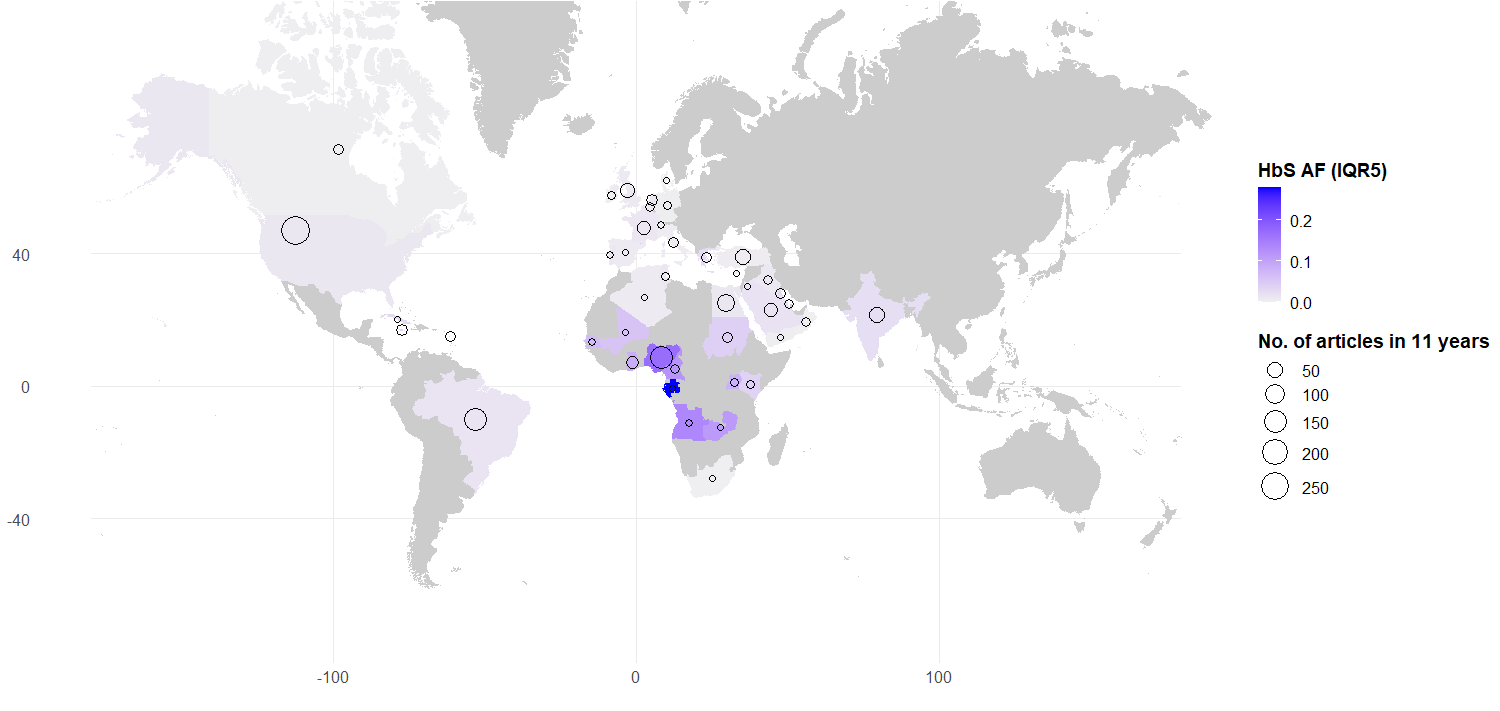

Supplement: Supplementary file 1 — Supplementary Material 1 [file 12874_2025_2513_MOESM1_ESM.docx]
